# Health Development versus Medical Relief: The Illusion versus the Irrelevance of Sustainability

**DOI:** 10.1371/journal.pmed.0030345

**Published:** 2006-08-15

**Authors:** Gorik Ooms

## Abstract

Ooms explores the ongoing "turf battle" between advocates of medical relief and advocates of health development.

Development agencies want their interventions to be sustainable. The Office of Sustainable Development of the Bureau for Africa of the United States Agency for International Development defines sustainability as “the ability of host country entities (community, public and/or private) to assume responsibility for programs and/or outcomes without adversely affecting the ability to maintain or continue program objectives or outcomes”. It defines financial sustainability as “having enough reliable funding”, meaning funding “generated from a country's own resources” [1].

Exceptionally, in times of emergencies, development agencies are willing to support relief interventions that are beyond the capacity of the beneficiaries. In such circumstances, sustainability is irrelevant, as the crisis is by definition temporary and the need for relief will ebb with the crisis.

In the field of health care, the issue of sustainability creates a dichotomy between medical relief and health development, because relief is unaffected by the condition of self-reliance. This dichotomy results in turf battles between the advocates of medical relief and the advocates of health development.

This essay explores the causes of this dichotomy and highlights the senselessness of turf battles. All trendy development approaches point out that sustainable health care—narrowly defined as independent from international aid—is illusionary in the world's poorest countries. Public health budgets in these countries must be increased and require stronger national and international financial commitments. If public health budgets were increased, the level of health care within the capacity of the beneficiaries would be higher, and the dichotomy would dissolve to a certain extent.

**Figure:**
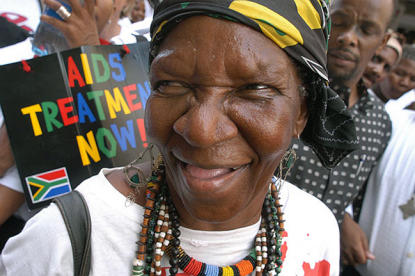
A rally to demand greater access to HIV medicines (Photo: Eric Miller)

However, health development advocates assume that public health budgets will not be increased and base their sustainability estimations on this assumption. In doing so, they contribute to the status quo of insufficient public health budgets.

## Turf Battles

### Funding of HIV medicines

In February 2000, exceptional rainfall inundated a part of southern Mozambique. Many people were rescued by helicopters. Providing helicopters was an unsustainable intervention, but many development agencies were willing to support it.

Mozambique is also a country highly affected by HIV/AIDS. Many people rescued with helicopters were HIV positive, and needed antiretroviral therapy (ART). Providing ART in Mozambique is also unsustainable. But Médecins Sans Frontières (MSF) considers the HIV/AIDS epidemic an emergency: MSF used helicopters to rescue people and planned to provide ART.

Development agencies initially refused to support the provision of ART in Mozambique. When the health ministry included ART pilot projects in the proposal for the Global Fund to Fight AIDS, Tuberculosis and Malaria, development agencies opposed ART funding. Not much of this opposition was formally recorded, but at least one of the agencies was outspoken: the United Kingdom Department for International Development (DFID) argued that “on the issue of anti-retroviral treatments, DFID would need to take a strong negotiating stand, as neither of the two predicted outcomes if ART were to be deployed by the GHF [Global Health Fund, the working title for the Global Fund to Fight AIDS, Tuberculosis and Malaria] are desirable, one resulting in a furthering of the inequalities in the distribution of health care in developing countries, the other would result in the rapid depletion of the GHF's budget” [2]. It should be noted that many development agencies opposed the introduction of ART; however, DFID is one of the agencies that has since reversed its opinion on the issue [3].

This turf battle is also fought in the medical literature. The position of health development advocates on HIV/AIDS has shifted from an emphasis on prevention [4,5] toward a balanced approach including both prevention and treatment [6]. However, in a recent article in *PLoS Medicine*, Rosen and colleagues promote the rationing of ART for the sake of sustainability [7]. Such rationing is a new formulation of the old notion that universal access to ART is unsustainable.

Peter Piot, the head of the Joint United Nations Programme on HIV/AIDS, referred to the relief approach when asking the World Bank and the International Monetary Fund to set aside their fiscal austerity. Piot compared countries affected by the HIV/AIDS epidemic with countries emerging from conflict: “For countries emerging from conflict, the Bank has pioneered a careful program of exceptions, running a calculated risk on the grounds that inaction would be riskier still. So let's now do something similar for AIDS, a risk far greater than conflict in many countries” [8].

The Southern Africa Humanitarian Information Management Network (hosted by the United Nations Office for the Coordination of Humanitarian Affairs, sponsored by several development agencies) is looking for a compromise: “The dichotomy of ‘humanitarian’ and ‘development’ assistance must be overcome; instead an approach should be composed of ‘developmental relief’ and ‘emergency development’” [9].

### Malnutrition

Exceptional levels of malnutrition constitute another example of turf battles. MSF considers malnutrition crises such as the recent one in Niger as emergencies, provides free health care, and condemns the “refusal to acknowledge the urgency of the situation and consider taking exceptional measures” [10].

Development agencies—often discreetly—reject this relief approach. The Direction Générale de la Coopération au Dévelopement (DGCD) of Belgium is an important donor to the public health budget of Niger, but it did not support MSF's request to consider taking exceptional measures in Niger. The strategy note of the DGCD on health care reveals why: the note says that free health care is unsustainable, that health insurance is the solution to make health care sustainable, and that free health care undermines the incentive to provide health insurance [11].

### Countries emerging from conflict

A third example of ongoing turf battles is related to the shift from relief to development in countries emerging from conflict. MSF considers the situation in Burundi to be a continuing crisis and advocates free health care [12]. Most development agencies, on the other hand, see Burundi as a country that should prepare for development; believing free health care to be unsustainable, these agencies do not support the abolition of user fees (fees levied on patients for receiving health services).

MSF published a report on epidemiological surveys in the Democratic Republic of Congo (DRC) [13]. The entire country, not just those regions affected by the conflict, is burdened by high mortality figures, and the MSF report recommends that health care should be subsidized so that it is available to patients free of charge. This recommendation seems to have fallen on deaf ears.

As it does for southern Africa, the United Nations Office for the Coordination of Humanitarian Affairs promotes a combination of relief and development for DRC. It considers DRC as a “chronic and unrelenting emergency” and confirms that “medical services, even when heavily subsidised, are beyond the reach of many citizens”. Nonetheless, it promotes “free-of-charge access to primary healthcare for IDPs [Internally Displaced Persons]/returnees and other groups at risk” but not for the general population [14]. Other agencies supporting health care in DRC, such as the World Bank, consider sustainability an important objective and promote “flexibility in terms of setting user fees and drug prices according to local circumstances and in a balanced manner” [15].

## The Illusion of Sustainability

The minimum budget required to finance adequate levels of health care in poor countries was estimated by the Commission on Macroeconomics and Health (CMH) to be US$35 per person per year [16]. Government expenditure on health in many countries stands far below this figure, with 33 countries in sub-Saharan Africa spending less than US$15 per person per year on health (Table 1).

**Table 1:**
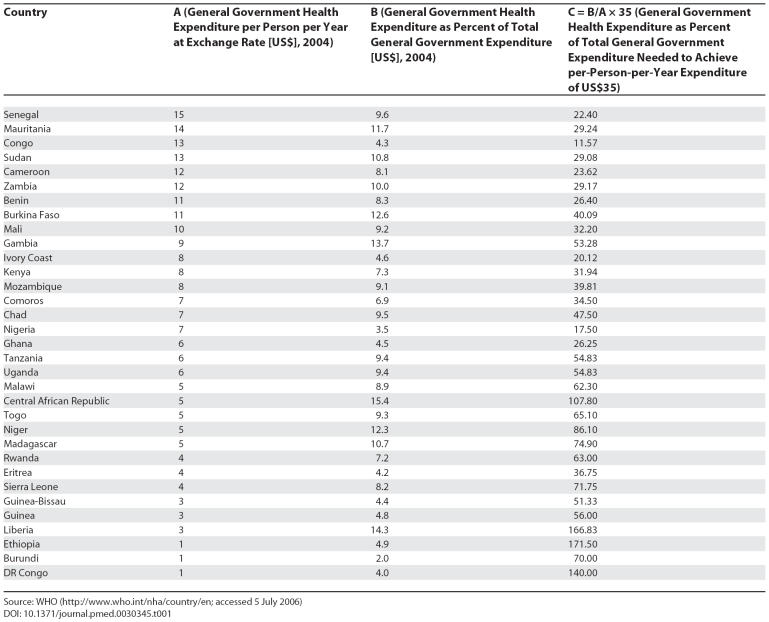
Countries in Sub-Saharan Africa Spending Less Than US$15 per Person per Year on Health

Some of these countries (Central African Republic, Liberia, Ethiopia, and DRC) would have to spend more than 100% of the overall government budget on health alone to achieve the US$35-per-person-per-year target. In 2004, only the Central African Republic was living up to the April 2001 “Abuja Declaration”, in which members of the Organisation of African Unity (which became the African Union) promised to allocate at least 15% of their annual budget to the improvement of the health sector [17].

The yearly amounts of additional international assistance needed to finance health care in poor countries were estimated by the CMH at US$22 billion per year by 2007 and US$31 billion per year by 2015. The governments of these countries would have to commit additional amounts of US$35 billion per year by 2007 and US$63 billion by 2015 to health care.

Development agencies continue to foster the illusion of sustainability.

World Bank experts estimate that it would cost between US$40 billion and US$70 billion in additional international assistance per year to realize the Millennium Development Goals (MDGs), which include some ambitious health-related goals [18]. The Millennium Development Project uses higher estimations: additional amounts of US$130 billion per year—which fit within the pledge of donor countries to allocate 0.7% of their gross national product to development assistance—would be “more than enough” [19].

The Committee on Economic, Social and Cultural Rights is mandated to monitor the achievements of governments with regards to the human rights included in the Covenant on Economic, Social and Cultural Rights, including the right to health. The committee insisted on the obligation to provide international assistance when it defined the absolute minimum level of health care: “For the avoidance of any doubt, the Committee wishes to emphasize that it is particularly incumbent on States parties and other actors in a position to assist, to provide ‘international assistance and cooperation, especially economic and technical’ which enable developing countries to fulfil their core and other obligations” [20]. The interventions on which the CMH based its estimations [21] fit within the minimum level of health care defined by the committee [22]. Development agencies endorsing health as a human right therefore endorse the obligation to provide additional international assistance to meet the US$35-per-person-per-year threshold.

No matter which of these approaches to health development one prefers, the conclusion is always the same: more national and international financial commitments to health care are needed, and sustainability—if narrowly defined as independent from international aid—is an illusion. The simple mathematical facts are that the public health budget of Niger would have to be multiplied by seven and the public health budgets of Burundi and DRC would have to be multiplied by 35 to achieve the US$35 threshold.

Nevertheless, development agencies continue to foster the illusion of sustainability [23]. When making their estimations, they turn a blind eye to the development approaches they promote elsewhere. The DGCD strategy note on primary health care endorses health as a human right, the MDGs, and the recommendations of the CMH [11]. Does the DGCD consider the consequences of these approaches as realistic options when it rejects free health care? It doesn't, which is very convenient for a governmental development agency. (Making the patients pay reduces the demand for health care and the overall costs for health ministries, and thus the level of international assistance needed.)

The World Bank remains ambivalent about health as a human right, but it does promote the MDGs. Does it consider the consequences of aiming for the MDGs in terms of financial assistance to the DRC public health budget as realistic options, when it promotes flexibility in terms of setting user fees and drug prices?

Rosen et al. “assume that donor support [for ART] will hold out for some time but will ultimately ebb, leaving national governments responsible for an increased share of the costs of treatment” [7]. Based on this assumption, they promote a way of providing ART that will exclude many, but one that will be sustainable for national governments. Prophecies about the shortage of international assistance can be self-fulfilling.

The self-fulfilling effect of prognoses about international assistance might also work in the opposite direction. When MSF started implementing comprehensive HIV/ AIDS interventions including ART, it assumed that more national and international financial resources for the fight against HIV/AIDS should and would become available. MSF rejected concerns about the sustainability of these interventions. The Organisation for Economic Co-operation and Development found that, between 1993 and 2003, international assistance directed toward health stagnated, except for interventions against HIV/ AIDS [24]. Rejecting concerns about sustainability might be the best way to defeat the illusion of sustainability and, paradoxically, to promote sustainability at a different level: the sustainability of international assistance.

## To Reinforce the Status Quo, or to Reject It?

Medical relief organisations are not driven by health development approaches (such as supporting health as a human right, achieving the MDGs, or investing in health care for economic growth). Instead, they are driven by a *humanitarian* impulse; there are overwhelming needs and there is an obligation to respond to those needs.

Development agencies do promote health as a human right, the MDGs, and investments in health care for economic growth. But when designing their interventions, these agencies assume that the financial means needed to realise the minimum level of the right to health, to achieve the MDGs, and to invest sufficiently in health care to promote economic growth will simply not be available. So they advocate the cheapest interventions and contribute to the status quo of insufficient public health budgets.

Health development advocates should not blame medical relief advocates for ignoring concerns about sustainability. The actions of medical relief agencies have nothing to do with ignorance; they are a deliberate choice. The status quo of insufficient public health budgets deserves only a firm rejection.

## Supporting Information

Text S1 Translation of Full Article into French by Author (101 KB DOC).

## Abbreviations

ART: antiretroviral therapy
CMH: Commission on Macroeconomics and Health
DFID: United Kingdom Department for International Development
DGCD: Direction Générale de la Coopération au Dévelopement
DRC: Democratic Republic of Congo
IDPs: internally displaced persons
MDGs: Millennium Development Goals
MSF: Médecins Sans Frontières
