# Supplementary material for: Health Development versus Medical Relief: The Illusion versus the Irrelevance of Sustainability
Source: PLoS Med. 2006 Aug 15;3(8):e345. doi: 10.1371/journal.pmed.0030345 (PMC1539094; doi:10.1371/journal.pmed.0030345)
Supplement: Supplementary file 1 [file pmed.0030345.sd001.doc]

# ESSAI

**Aide au développement ou aide médicale d'urgence.**

La durabilité: illusoire ou non pertinente

Gorik Ooms

Gorik Ooms est directeur général de la section belge de Médecins Sans Frontières.

E-mail: [gorik.ooms@brussels.msf.org](mailto:gorik.ooms@brussels.msf.org)

Financement : Gorik Ooms n’a bénéficié d’aucun financement particulier pour écrire cet article.

Intérêts concurrentiels : Médecins Sans Frontières est une association humanitaire médicale privée.

Copyright: © 2006 Gorik Ooms. Le présent article est en libre accès et sa distribution est soumise à la « Creative Commons Attribution Licence », qui permet, sans restriction, son utilisation, sa distribution et sa reproduction sur n’importe quel média, à la condition de l’attribuer à son auteur original et de citer la source.

**Abréviations** : ARV, traitement antirétroviral ; CMH, Commission Macroéconomie et Santé; DFID, Département pour le développement international du Royaume Uni; DGCD, Direction Générale de la Coopération au Développement; RDC, République Démocratique du Congo; OMD, Objectifs du Millénaire pour le Développement; MSF, Médecins Sans Frontières

Les agences de coopération au développement souhaitent appuyer des interventions qui s'inscriront dans une logique de durabilité et de pérennité. Le Bureau de l’Afrique pour le développement durable de l’Agence des Etats-Unis pour le développement international (USAID) définit la durabilité comme « la capacité des entités (communautaires, publiques et/ou privées) des pays bénéficiaires à assumer la responsabilité de programmes et/ou de leurs résultats sans porter atteinte à la capacité de maintenir ou de poursuivre les objectifs ou les résultats des programmes. » La durabilité financière y est décrite par le fait de « disposer de fonds fiables suffisants », entendant par là des fonds « provenant des ressources propres du pays ». ^[1]^

Exceptionnellement, en période d'urgence, les agences de coopération sont prêtes à participer à des interventions humanitaires et à déployer des moyens supérieurs à la capacité des bénéficiaires. De telles interventions ne s’inscrivent alors pas dans une perspective de durabilité puisque, par définition, la crise est temporaire et que les besoins humanitaires disparaîtront avec la fin de la crise.

La question de la durabilité crée une dichotomie entre d'un côté l'aide médicale d'urgence et de l'autre, l’aide au développement, l'aide humanitaire n'étant pas soumise à des critères imposant la recherche de l'autonomie des populations assistées. Cette dichotomie provoque des querelles de clocher entre défenseurs de l'aide médicale d'urgence et défenseurs de l’aide au développement dans le domaine des soins de santé.

Cet essai tente d’examiner les causes de cette dichotomie et souligne l'absurdité de ces querelles de clocher. Les approches récentes dans le domaine de la coopération au développement illustrent bien le fait qu'il est illusoire de penser que, dans les pays les plus pauvres, les soins de santé puissent être durables - à savoir non soutenus par l’aide internationale. Les budgets affectés à la santé publique dans ces pays doivent être augmentés et nécessitent des engagements financiers nationaux et internationaux plus solides. Si des budgets plus importants étaient alloués à la santé publique, les bénéficiaires auraient les moyens de se donner un niveau de santé plus élevé, et la dichotomie disparaîtrait partiellement.

Mais les défenseurs du développement en matière de soins de santé supposent que les budgets ne seront pas augmentés et se fondent sur cette supposition pour leurs évaluations de durabilité. Ils contribuent ainsi au statu quo de l’insuffisance des budgets affectés à la santé publique.

### Querelles de clocher

**Le financement des médicaments contre le VIH/Sida.** En février 2000, des pluies d’une intensité exceptionnelle ont provoqué des inondations dans le sud du Mozambique. De nombreuses personnes ont été secourues par hélicoptère. L’acheminement d’hélicoptères était une intervention non durable mais nombre d’agences de développement ont participé à ces interventions et en ont assumé les frais.

Le Mozambique est également un pays fortement touché par le VIH/sida. Parmi les personnes secourues par hélicoptère, bon nombre étaient séropositives et avaient besoin d’un traitement antirétroviral (ARV). La prise en charge du traitement des patients Sida au Mozambique ne répond pas non plus aux critères de durabilité. Médecins Sans Frontières considère que l'épidémie de Sida au Mozambique constitue également une urgence: nous avons donc utilisé des hélicoptères pour venir en aide à la population et décidé de mettre en place des projets de traitement sida.

Les agences de coopération au développement ont d’abord refusé d’apporter leur soutien à la prise en charge du traitement Sida au Mozambique. Lorsque le ministre de la santé a inclus des projets pilotes relatifs aux ARV dans la proposition pour le Fonds mondial de lutte contre le sida, la tuberculose et le paludisme, les agences de développement se sont même opposées au financement de ces médicaments. Ces oppositions n'ont pas fait l'objet de grandes déclarations publiques, mais l'une de ces agences s'est positionnée très clairement sur ce point: le Department For International Development du gouvernement britannique (DFID) a fait savoir que: « sur la question des antirétroviraux, DFID devait adopter une position de négociation très ferme sachant que de son point de vue le fait que le Fonds Mondial de lutte contre le Sida, la tuberculose et la malaria appuie la distribution d'antirétroviraux aboutirait à deux résultats non souhaités: l'extension des inégalités dans la délivrance des soins de santé dans les pays en développement et la réduction rapide des budgets disponibles via le Fonds Global ». ^[2]^ Il est à noter qu'historiquement la grande majorité des agences de développement s’est opposée à l'introduction des ARV. DFID est une des agences qui a radicalement changé de point de vue depuis lors. ^[3]^

Ces oppositions sont également perceptibles dans la littérature médicale. La position des défenseurs de l’aide au développement dans le domaine de la santé a évolué sur la question du VIH-Sida. Ils ont cessé de défendre une intervention axée exclusivement sur la prévention ^[4],^ ^[5]^, appuyant aujourd'hui une approche équilibrée entre l'aide à la prévention et au traitement. ^[6]^ Toutefois, S. Rosen et collaborateurs, dans un article récent de *PLoS Medicine*, encouragent le rationnement des ARV par souci de durabilité. ^[7]^ L'idée de rationner l'accès aux antirétroviraux dans les pays en développement n’est rien d’autre en réalité que la résurgence de cette fameuse croyance selon laquelle l'accès universel aux traitements Sida ne s'inscrit pas dans la durabilité.

Peter Piot, Directeur du Programme des Nations Unis sur le VIH-SIDA a défendu la logique d'urgence de la lutte contre le Sida en appelant la Banque Mondiale et le Fonds Monétaire International à abandonner la politique d’austérité fiscale qu’ils menaient jusque là. En effet, pour Peter Piot les pays touchés par l’épidémie de VIH/sida sont comparables aux pays sortant d'une période de conflit : « Lorsqu’un pays sort d’un conflit, la Banque avance avec prudence un programme d’exceptions; elle prend ce risque calculé en se disant que l’inaction constituerait un risque probablement plus important encore. Il en va de même pour le sida, qui, dans beaucoup de pays, représente un risque autrement plus élevé que le risque de conflit ». ^[8]^

Le Réseau sud africain d'informations humanitaires pour une réponse coordonnée aux désastres—projet du Bureau des Nations Unies pour la Coordination des Affaires Humanitaires et sponsorisé par plusieurs agences de coopération au développement—recherche quant à lui un compromis : « la dichotomie entre aide humanitaire et aide au développement doit être surpassée pour aller vers une approche d'action humanitaire prenant en compte le développement et une approche de développement prenant en compte l’humanitaire ». ^[9]^

**La malnutrition.** Les taux exceptionnels de malnutrition nous fournissent un autre exemple illustratif de cette opposition autour des critères de durabilité. MSF considère comme des urgences les taux de malnutrition affichés aujourd'hui dans plusieurs régions du Niger. Et en ce sens, MSF fournit gratuitement des soins de santé aux populations à qui nous venons en aide. Notre association a condamné publiquement « le refus de reconnaître l'urgence de la situation au Niger et la nécessité de prendre des mesures exceptionnelles ». ^[10]^

Les agences de coopération au développement, souvent discrètement, rejettent l’approche axée sur l’aide d’urgence. La Direction Générale de la Coopération au Développement (DGCD) de Belgique consacre des budgets importants pour la santé publique du Niger mais elle n’a pas soutenu la demande de MSF d'envisager des mesures exceptionnelles au Niger. La note de stratégie de la DGCD sur les soins de santé motive son refus de la sorte: la gratuité des soins de santé n’est pas durable; pour rendre les soins de santé durables, il faut envisager un système d’assurance maladie or, la gratuité des soins de santé n’encourage pas l’affiliation aux assurances. ^[11]^

**Les pays sortant de conflits.** Une troisième illustration de ce point de discorde est liée à la transition entre aide d’urgence et aide au développement dans les pays sortant d’un conflit. Pour MSF, le Burundi est un pays qui connaît toujours une situation de crise et l’organisation y défend donc des soins de santé gratuits. ^[12]^ En revanche, la plupart des agences de coopération au développement considèrent le Burundi comme un pays qui doit se préparer au développement. Ces agences qui considèrent dès lors les soins de santé gratuits comme non durables, sont donc opposées à la suppression des soins payants.

MSF a publié un rapport sur des études épidémiologiques en République démocratique du Congo (RDC). ^[13]^ Il ressort de ce rapport que tout le pays, et pas seulement les régions affectées par le conflit, enregistre des taux de mortalité élevés. Le rapport de MSF préconise donc des soins de santé subsidiés et gratuits pour tous les patients mais il semblerait que cette recommandation soit tombée dans l’oreille d’un sourd.

OCHA, comme il le fait pour l’Afrique du Sud, prône la combinaison d’aide d’urgence et de développement en RDC. OCHA considère la RDC comme une « urgence chronique et continue » et confirme que « pour de nombreux d’habitants, les services médicaux, même lorsqu’ils sont largement subsidiés, restent trop onéreux et hors de portée ». Pourtant, s’il encourage « l’accès gratuit aux soins de santé primaire pour les personnes déplacées à l’intérieur du pays ou rapatriées ainsi que pour les autres groupes à risque », il n’y inclut pas la population générale. ^[14]^ D’autres agences qui apportent leur soutien aux soins de santé en RDC, telles que la Banque mondiale, considèrent la durabilité comme un objectif important et encouragent « la flexibilité dans la fixation de la contribution du patient et des prix des médicaments en fonction des circonstances locales et selon une approche équilibrée ». ^[15]^

### Illusoire durabilité

La Commission Macroéconomie et Santé (CMH) a estimé à 35 dollars par habitant et par an le budget minimum requis pour financer, dans les pays pauvres, des soins de santé à un niveau adéquat. ^[16]^ Dans beaucoup de pays, les dépenses de santé des gouvernements sont bien en deçà de ce chiffre, et, dans 33 pays au moins d’Afrique sub-saharienne, le budget consacré au secteur de la santé est inférieur à 15 dollars par personne et par an (Tableau 1).

Pour atteindre cet objectif de 35 dollars par habitant et par an, certains de ces pays (République centreafricaine, Ethiopie et RDC) devraient consacrer plus de 100% du budget total du gouvernement rien qu’à la santé. En 2004, seule la République centreafricaine respectait la Déclaration d’Abuja, adoptée en avril 2001, dans laquelle les membres de l’Organisation de l’Unité Africaine (transformée en Union Africaine) promettent de consacrer au moins 15% de leur budget annuel à l’amélioration du secteur des soins de santé. ^[17]^

La CMH a estimé à 22 milliards de dollars par an d’ici 2007 et à 31 milliards de dollars par an d’ici 2015, les sommes supplémentaires d’aide internationale à consentir au financement des soins de santé dans les pays pauvres. Les gouvernements de ces pays devraient affecter au secteur de la santé des sommes supplémentaires à concurrence de 35 milliards par an d’ici 2007 et de 63 milliards par an d’ici 2015.

Les experts de la Banque Mondiale estiment que l’aide internationale supplémentaire devrait être de 40 à 70 milliards de dollars par an afin d’atteindre les Objectifs du Millénaire pour le Développement (OMD), qui comportent certains objectifs ambitieux dans le domaine de la santé. ^[18]^ Le Projet de développement du Millénaire utilise des estimations plus élevées : un effort supplémentaire pour un montant de 130 milliards de dollars par an—ce qui entre dans les promesses des pays donateurs de consacrer 0,7% de leur produit national brut à la coopération au développement— serait « plus que suffisant ». ^[19]^

Le Comité des Droits Economiques, Sociaux et Culturels a pour mission de contrôler les réalisations accomplies par les gouvernements en matière des droits de l’homme reconnus dans le Pacte international relatif aux droits économiques, sociaux et culturels, notamment le droit à la santé. Dans sa définition du niveau minimum absolu en matière de soins de santé, le Comité insiste sur l’obligation de fournir une aide internationale : « Pour qu'il n'y ait aucun doute à ce sujet, le Comité tient à souligner qu'il incombe tout particulièrement aux États parties et aux autres intervenants en mesure d'apporter leur concours de fournir l'assistance et la coopération internationales, notamment sur les plans économique et technique, nécessaires pour permettre aux pays en développement d'honorer les obligations fondamentales et autres ». ^[20]^  Les interventions sur lesquelles le CMH base ses estimations ^[21]^ répondent au paquet minimum de soins de santé défini par ce même Comité. ^[22]^ Les agences de coopération au développement qui comptent la santé comme un droit de l’homme souscrivent, par conséquent, à l’obligation d’aide internationale supplémentaire et au seuil de 35 dollars par personne et par an.

Quelle que soit l’approche de l’aide au développement dans le domaine de la santé que l’on retienne, la conclusion est toujours la même: les efforts financiers nationaux et internationaux pour les soins de santé doivent être plus importants, et la durabilité financière—à savoir hors soutien financier international—est une illusion. Les calculs sont simples : le budget de la santé publique du Niger devrait être multiplié par sept et ceux du Burundi et de la RDC par 35 pour atteindre le seuil des 35 dollars.

Malgré tout, les agences de développement continuent de nourrir l’illusion de la durabilité. ^[23]^ Dans la pratique de leurs estimations et interventions, elle font fi des principes de développement qu’elles promeuvent par ailleurs. La note de stratégie de la DGCD sur les soins de santé primaires considère la santé comme un droit de l’homme, appuie les objectifs du Millénaire et les recommandations de la CMH. ^[24]^ La DGCD envisage-t-elle comme des choix réalistes les conséquences de ces approches lorsqu’elle rejette la gratuité des soins de santé ? La réponse est non et ceci semble être bien commode pour une agence de coopération au développement (si les patients doivent payer, la demande en soins de santé diminue, de même que le coût total pour les ministères de la santé, et donc le niveau de l’aide internationale requise).

La Banque Mondiale reste ambivalente au sujet de la santé en tant que droit de l’homme mais elle encourage les OMD. Envisage-t-elle les conséquences de la poursuite des OMD au plan de l’aide financière dans le budget de la santé publique de la RDC comme des options réalistes lorsqu’elle favorise la flexibilité pour la fixation des prix des soins et des médicaments à payer par les patients ?

Rosen *et al*. « supposent que l’aide des Bailleurs de fonds [pour les ARV] durera un petit temps mais finira par se tarir, laissant aux gouvernements internationaux la responsabilité d’augmenter leur contribution dans le financement des traitements». ^[25]^ À partir de cette supposition, les gouvernements nationaux favoriseront la mise en place d’un système durable pour la fourniture d’ARV tout en excluant de nombreuses personnes de ce système. Les prophéties sur le manque d’aide internationale provoquent elles-mêmes ce qu’elles annoncent.

Mais les pronostics pourraient produire l’effet voulu sur l’aide internationale dans l’autre sens également. Lorsque MSF a commencé à offrir des soins intégrés contre le VIH/sida, incluant les ARV, elle supposait que les ressources financières nationales et internationales pour la lutte contre cette maladie devaient être plus importantes et le deviendraient. MSF a rejeté les préoccupations concernant la durabilité de ces interventions. L’Organisation de coopération et de développement économiques (OCDE) a trouvé qu’entre 1993 et 2003, l’aide internationale destinée à la santé n’a pas augmenté si ce n’est pour la lutte contre le VIH/sida. **^[26]^** Le rejet des préoccupations de durabilité pourrait constituer la meilleure manière de déforcer cette illusion et, paradoxalement, de favoriser la durabilité à un autre niveau : la durabilité de l’aide internationale.

Renforcement ou rejet du statu quo

L’action des organisations médicales humanitaires n’est pas guidée par les principes qui sous–tendent l’aide au développement (qu’il s’agisse de la promotion de la santé comme droit de l’homme, du soutien des objectifs du Millénaire ou de l’investissement dans la santé pour soutenir la croissance économique). Leurs interventions sont dictées par la motivation d’agir face à des besoins jugés importants et l’obligation d’y apporter une réponse.

Les agences de développement quant à elles promeuvent la santé en tant que droit de l’homme et encouragent les OMD et les investissements dans les soins de santé en vue de la croissance économique. Mais, pour l’élaboration de leurs interventions, ces agences prennent comme a priori l’insuffisance des moyens financiers pour garantir le paquet minimum de droit à la santé, la réalisation des objectifs du Millénaire et l’investissement nécessaire dans le domaine de la santé en tant que gage de développement économique. En conséquence, ces agences défendent le principe d’intervention aux coûts contrôlés et par là même contribue au maintien d’une insuffisance des budgets alloués à la santé publique.

Les défenseurs de l’aide au développement dans le secteur de la santé ne devrait pas reprocher aux défenseurs de l’aide médicale d’urgence la non prise en compte du critère de durabilité ; les actions des organisation médicales humanitaires ne résultent pas d’une ignorance mais bel et bien d’un choix pesé et délibéré. Quand à l’acceptation de l’insuffisance des budgets publiques alloués à la santé publique, elle doit être réfutée.

Tableau 1. Pays d’Afrique sub-saharienne consacrant à la santé moins de 15 dollars par personne et par an.

| Pays | A  Dépenses publiques de santé par habitant au taux de change officiel [US $], 2004 | B  Dépenses publiques de santé en % du total des dépenses publiques, [US $], 2004 | C = B/A * 35  Dépenses publiques de santé en % du total des dépenses publiques, nécessaires pour atteindre une dépense de santé de 35 dollars par habitant par an |
| --- | --- | --- | --- |
| Sénégal | 15 | 9.6 | 22.40 |
| Mauritanie | 14 | 11.7 | 29.24 |
| Congo | 13 | 4.3 | 11.57 |
| Soudan | 13 | 10.8 | 29.08 |
| Cameroun | 12 | 8.1 | 23.62 |
| Zambie | 12 | 10.0 | 29.17 |
| Benin | 11 | 8.3 | 26.40 |
| Burkina Faso | 11 | 12.6 | 40.09 |
| Mali | 10 | 9.2 | 32.20 |
| Gambie | 9 | 13.7 | 53.28 |
| Côte d’Ivoire | 8 | 4.6 | 20.12 |
| Kenya | 8 | 7.3 | 31.94 |
| Mozambique | 8 | 9.1 | 39.81 |
| Comores | 7 | 6.9 | 34.50 |
| Chad | 7 | 9.5 | 47.50 |
| Nigeria | 7 | 3.5 | 17.50 |
| Ghana | 6 | 4.5 | 26.25 |
| Tanzanie | 6 | 9.4 | 54.83 |
| Ouganda | 6 | 9.4 | 54.83 |
| Malawi | 5 | 8.9 | 62.30 |
| République Centrafricaine | 5 | 15.4 | 107.80 |
| Togo | 5 | 9.3 | 65.10 |
| Niger | 5 | 12.3 | 86.10 |
| Madagascar | 5 | 10.7 | 74.90 |
| Rwanda | 4 | 7.2 | 63.00 |
| Erythrée | 4 | 4.2 | 36.75 |
| Sierra Leone | 4 | 8.2 | 71.75 |
| Guinée-Bissau | 3 | 4.4 | 51.33 |
| Guinée | 3 | 4.8 | 56.00 |
| Libéria | 3 | 14.3 | 166.83 |
| Ethiopie | 1 | 4.9 | 171.50 |
| Burundi | 1 | 2.0 | 70.00 |
| République Démocratique du Congo | 1 | 4.0 | 140.00 |

Source: WHO (<http://www.who.int/nha/country/en>; consulté le 5 juillet 2006)

1. Mehdi S (1999) Health and Family Planning Indicators: Measuring Sustainability. Washington: USAID, Office of Sustainable Development, Bureau for Africa. <http://sara.aed.org/publications/cross_cutting/indicators/indicators2.pdf> consulté le 23 juin 2006
2. DFID (2001) Minutes of the Development Committee Meeting, 20 September 2001. <http://www.dfid.gov.uk/aboutdfid/dev-committee/dcmeeting20sept01.asp> consulté le 23 juin 2006
3. DFID (2004) Taking action. The UK’s strategy for tackling HIV and AIDS in the developing world. London: DFID. <http://www.dfid.gov.uk/pubs/files/hivaidstakingaction.pdf> consulté le 23 juin 2006
4. Creese A, Royd K, Alban A & Guiness L (2002) Cost-effectiveness of HIV/AIDS interventions in Africa: a systematic review of the evidence. Lancet 359: 1635
5. Marseille E, Hofmann P & Kahn J (2002) HIV prevention before HAART in sub-Saharan Africa. Lancet 359: 1851
6. Salomon J, Hogan D, Stover J, Stanecki K, Walker N, Ghys P, et al. (2005) Integrating HIV Prevention and Treatment: From Slogans to Impact. PLoS Med 2(1): e50. <http://medicine.plosjournals.org/archive/1549-1676/2/1/pdf/10.1371_journal.pmed.0020016-L.pdf> consulté le 23 juin 2006
7. Rosen S, Sanne I, Collier A, Simon J (2005) Rationing Antiretroviral Therapy for HIV/AIDS in Africa: Choices and Consequences. PloS Med 2(11): e303 <http://medicine.plosjournals.org/archive/1549-1676/2/11/pdf/10.1371_journal.pmed.0020303-L.pdf> consulté le 23 juin 2006
8. Piot P (2003) The Need For An Exceptional Response To An Unprecedented Crisis. Presidentials Fellows Lecture. Washington: World Bank. <http://web.worldbank.org/WBSITE/EXTERNAL/NEWS/0,,contentMDK:20140527~menuPK:34476~pagePK:34370~piPK:34424~theSitePK:4607,00.html> consulté le 23 juin 2006
9. SAHIM (2005) Inter Agency Regional Strategic Framework for Southern Africa. Johannesburg: Sahim. <http://www.sahims.net/doclibrary/Sahims_Documents/050613_och1.pdf> consulté le 23 juin 2006
10. MSF (2005) Niger Food Crisis: Early Diagnosis, Slow and Misguided Aid. News Update, 23 June 2005. Paris: MSF. <http://www.doctorswithoutborders.org/news/2005/06-23-2005.cfm> consulté le 23 juin 2006
11. DGCD. (2002) Note Stratégique: Soins de Santé Primaires. Brussels: DGCD. <http://www.dgos.be/documents/fr/notes_strategiques/sante/note_sante_fr.pdf> consulté le 23 juin 2006
12. Philips M, Vazquez I, Sprecher A (2005) Good donorship in practice: the case of Burundi. Humanitarian Exchange 29 March 2005: 21 <http://www.odihpn.org/documents/humanitarianexchange029.pdf> consulté le 23 juin 2006
13. MSF (2005) Access to Health Care, Mortality and Violence in Democratic Republic of the Congo. Brussels: MSF. <http://www.doctorswithoutborders.org/publications/reports/2005/drc_healthcare_11-2005.pdf> consulté le 23 juin 2006
14. Bureau des Nations Unies pour la coordination des affaires humanitaires (2006) Plan d’action 2006 République démocratique du Congo. Kinshasa: Bureau des Nations Unies pour la coordination des affaires humanitaires.

    <http://www.rdc-humanitaire.net/planaction/plan2006.htm> consulté le 23 juin 2006
15. World Bank (2005) Health Sector Rehabilitation Support Project. Project Appraisal Document. Washington: World Bank. <http://www-wds.worldbank.org/servlet/WDSContentServer/WDSP/IB/2005/08/10/000090341_20050810100414/Rendered/PDF/30855.pdf> consulté le 23 juin 2006
16. CMH (2001) Macroeconomics and Health: Investing in Health for Economic Development. Report of the Commission on Macroeconomics and Health. Geneva: WHO. <http://www.cid.harvard.edu/cidcmh/CMHReport.pdf> consulté le 23 juin 2006
17. OAU (2001) Abuja Declaration on HIV/AIDS, Tuberculosis and Other Related Infectious Diseases. Abuja: OAU [http://www.uneca.org/adf2000/Abuja%20Declaration.htm](http://www.uneca.org/adf2000/Abuja Declaration.htm) consulté le 23 juin 2006
18. Devaranjan S, Miller M & Swanson E (2002) Goals for Development: History, Prospects, and Costs. Washington: World Bank. <http://wdsbeta.worldbank.org/external/default/WDSContentServer/IW3P/IB/2002/04/26/000094946_02041804272578/Rendered/PDF/multi0page.pdf> consulté le 23 juin 2006
19. Sachs J & McArthur J (2005) The Millennium Project: a plan for meeting the Millennium Development Goals. Lancet 365: 347
20. CESCR (2000) The right to the highest attainable standard of health: CESCR General Comment 14. Geneva: Office of the United Nations Commissioner for Human Rights.
21. CMH (2001) Costs of scaling up Priority Health Interventions in Low-income and selected Middle-income Countries: Methodology and Estimates. Geneva: WHO. <http://www.cmhealth.org/docs/wg5_paper19.pdf> consulté le 23 juin 2006
22. Hammonds R & Ooms G (2004) World Bank Policies and the Obligation of Its Members to Respect, Protect and Fulfil the Right to Health. Health and Human Rights 8(1): 26
23. Kremer M & Miguel E (2004) The Illusion of Sustainability. CID Working Paper No. 112. Cambridge: Center for International Development at Harvard University. <http://www.ksg.harvard.edu/cid/cidwp/pdf/112.pdf> consulté le 23 juin 2006
24. DGCD. (2002) Note Stratégique: Soins de Santé Primaires. Brussels, DGCD. <http://www.dgos.be/documents/fr/notes_strategiques/sante/note_sante_fr.pdf> consulté le 23 juin 2006
25. Rosen S, Sanne I, Collier A, Simon J (2005) Rationing Antiretroviral Therapy for HIV/AIDS in Africa: Choices and Consequences. PloS Med 2(11): e303 <http://medicine.plosjournals.org/archive/1549-1676/2/11/pdf/10.1371_journal.pmed.0020303-L.pdf> consulté le 23 juin 2006
26. MacKellar L (2005) Priorities in global assistance for health, AIDS and population. Paris: Organisation for Economic Co-operation and Development. <http://www.oecd.org/dataoecd/42/39/34987795.pdf> consulté le 23 juin 2006.
